# Proceedings of the Eleventh International Meeting on Neuroacanthocytosis Syndromes

**DOI:** 10.5334/tohm.826

**Published:** 2023-11-03

**Authors:** Lars Kaestner

**Affiliations:** 1 Theoretical Medicine and Biosciences, Saarland University, Campus University Hospital, 66421 Homburg/Saar, Germany; 2 Dynamics of Fluids, Experimental Physics, Saarland University, 66123 Saarbruecken, Germany

**Keywords:** McLeod syndrome, XK-disease, Chorea Acanthocytosis, VPS13A-disease, bridge-like lipid transfer proteins, bulk lipid transfer disorders

## Abstract

The 11^th^ International Meeting on Neuroacanthocytosis Syndromes was held on September 15^th^–17^th^, 2023 at the University Hospital Campus in Homburg/Saar, Germany. The meeting followed the previous ten international symposia, the last of which was held online due to restrictions due to COVID19, in March 2021. The setting of the meeting encouraged interactions, exchange of ideas, and networking opportunities among the participants from around the globe, including basic and clinical scientists, clinicians, and especially patients, their relatives and caregivers. A total of about 20 oral communications were presented in five scientific sessions accompanied by a keynote lecture, a “Poster-Blitz” session, the “Glenn Irvine Prize” lecture and a panel discussion about “Patient registries, international cooperation & future perspectives”.

In summary, attendees discussed recent advances and set the basis for the next steps, action points, and future studies in close collaboration with the patient associations, which were actively involved in the whole process.

## Introduction

VPS13A disease (formerly chorea-acanthocytosis; due to bi-allelic mutations in *VPS13A*) and XK disease (formerly McLeod syndrome; due to mutations of *XK*) are rare neurodegenerative diseases, classified together as the “neuroacanthocytosis syndromes” [1]. In addition to neurological features, deformed red blood cells in the peripheral blood, known as acanthocytes, give the syndrome its name. Progressive neuronal degeneration of the basal ganglia, especially the caudate nucleus, is seen in neuroimaging and neuropathology. The clinical picture can resemble Huntington’s disease and can involve a broad spectrum of various movement disorders, including chorea, tics, dystonia, and parkinsonism. Other typical features are seizures, peripheral neuromyopathy, cognitive decline, and behavioral changes. Disease onset is typically in young-mid adulthood (VPS13A disease) or middle age (XK disease). Both diseases are chronic and progressive, resulting in dependence on activities of daily living and shortened life expectancy. Preliminary studies of post-mortem human brain tissue suggest lipid metabolism dysfunction at the subcellular level.

The 11^th^ International Meeting on Neuroacanthocytosis Syndromes was held on September 15^th^–17^th^, 2023, at the campus of the Saarland University Hospital of Saarland University at Homburg/Saar, Germany. The conference followed the tradition of the previous ten international symposia; the last in-person meeting was the 9^th^ meeting held in Dresden, Germany in March 2018 [2], followed by an online meeting in March 2021 organized by colleagues from Barcelona [3].

The conference focused primarily on the disease-related proteins, VPS13A and XK, which are involved in bulk lipid transfer at membrane contact sites. They closely interact forming a protein complex at the contact sites between the endoplasmic reticulum and the cellular membrane system. VPS13A and the other VPS13 proteins (VPS13B-D) belong to the newly acknowledged superfamily of bridge-like lipid transfer proteins (BLTPs). Mutations of the other *VPS13* genes cause either neurodegenerative (*VPS13C, D*) or neurodevelopmental (*VPS13B*) disorders. While the underlying pathophysiology is not yet known, it is possible that VPS13A and XK diseases, as well as the related conditions, are part of a new group of disorders with a common mechanism of impaired bulk lipid transport.

A total of approximately 20 oral communications were presented in five scientific sessions, accompanied by a keynote lecture by Prof. Adrian Danek entitled “Neuroacanthocytosis syndromes at 70: a term to be retired?” (abstract below). A “Poster-Blitz” session introduced the top seven posters. The 2023 “Glenn Irvine Prize” was given to Dr. Kevin Peikert who gave the prize-associated lecture (abstract below). The panel discussion about “Patient registries, international cooperation & future perspectives” continued the positive developments of the previous meetings regarding the participation and contributions of patients, their families, and caregivers.

A special session regarding red blood cell-related research was a joint session between the 11^th^ International Meeting on Neuroacanthocytosis Syndromes and the “EVIDENCE” project, a doctorate training program funded by the European Community. Further funding bodies include the German Research Foundation (DFG) the Greater Region (consisting of ‘Lorraine’ in France, ‘Wallonia’ in Belgium, ‘Saarland’ and ‘Rhineland-Palatinate’ in Germany as well as ‘Luxembourg’) and the Karlsberg Brewery (Homburg/Saar).

## Keynote lecture

**Neuroacanthocytosis syndromes at 70: a term to be retired?**

*Adrian Danek*

Department of Neurology, LMU University Hospital, LMU Munich, Munich, Germany

Unequivocal terms are a prerequisite for confronting the world and communicating about it. In 1952, Karl Singer, a hematologist from Vienna and Chicago, introduced the Greek word for thorn/prickle (ἄκανθα) to describe an as yet novel appearance of red cells. The associated disease “acanthocytosis” (of Bassen and Kornzweig) was quickly renamed to abetalipoproteinemia, once its biomarker signature had been discovered.

As the peculiar “thorny” erythrocyte shape was observed also with normal lipoproteins, the existence of further conditions of acanthocytosis (now turned into a mere finding and no longer a singular disease entity) became obvious. Apart from the occurrence in severe liver disease (with “spur cell” as alternative designation for the erythrocyte deformation), various neurological findings were found in association with acanthocytosis. These cases were designated to suffer from neuroacanthocytosis or Levine-Critchley syndrome. The often-quoted series of Hardie et al. (1991), however, has since been found to be genetically heterogeneous. This series contains cases due to mutations in *XK, VPS13A*, and *PANK2*. In addition, the index families of Levine et al. (1968) and of Critchley et al. (1968) are now known to harbor mutations of *XK* and of *VPS13A*, respectively.

“Neuroacanthocytosis” retains some usefulness (1) as a tentative label while working up a complex case, (2) as an umbrella term to group conditions that are related because their molecular substrates interact (as shown for the XK/McLeod and chorein/VPS13A proteins), and (3) as a tool for advocating for patients and their carers. “Neuroacanthocytosis”, however, does not represent a final medical diagnosis and must be shunned if precision medicine is the goal. Further, its continued use perpetuates the problem of constructing and imagining an entity based on a mere word – as not all patients with *VPS13A* and *XK* mutations present with acanthocytosis or with neurological findings, diagnostic delays result from fixation on the outdated term. The terms “XK disease”, “VPS13A disease”, and “PKAN” – even if somewhat “bloodless” – are superior if personalized medicine and, in particular, personalized treatment is pursued. The individual patient with one of these rare and ultra-rare diseases deserves a definite, genetically based diagnosis.

## Bridge-Like Lipid Transfer Proteins (BLTP) – a newly acknowledged protein super-family

**The notorious RBG proteins: long, thin and now with more FFAT**

*Tim Levine*

University College London, Institute of Ophthalmology, London, UK

VPS13A is one of ten human bridge-like lipid transfer proteins. The VPS13A bridge is assembled from 12 repeating beta-groove domains, each one elongating the bridge, which ends up long enough to span from one intracellular compartment to another. One surface of the bridge is entirely hydrophobic, allowing lipid molecules to slide along its full length. Now we know these bridges exist, a follow-on question is to find out which compartments they bridge between. It has already been shown that VPS13A bridges from the endoplasmic reticulum to various other compartments. This is made possible by the presence of a FFAT motif in VPS13A, and similar motifs are found in its two closest homologues VPS13C and VPS13D. Here we have used bioinformatics approaches to determine if other bridge-like lipid transfer proteins across evolution also contain FFAT motifs, which would indicate that they too have specific relationships with the endoplasmic reticulum.

**Facts and open questions about VPS13 family proteins**

*Pietro De Camilli*

Departments of Neuroscience and Cell Biology, Howard Hughes Medical Institute, Program in Cellular Neuroscience, Neurodegeneration and Repair, Yale University School of Medicine, New Haven, CT06510, USA

The discovery that VPS13 family proteins are lipid transport proteins (LTPs), and more specifically proteins that transport lipids by a bridge-like mechanism at membrane contact sites, has opened the possibility of addressing mechanistic questions about mechanisms of VPS13A disease. VPS13A was initially detected at contacts between the ER and mitochondria and, at least in cells overexpressing it, also at contacts between the ER and lipid droplets. However, it has subsequently been learned that VPS3A also functions at other membrane contacts, ER-plasma membrane contacts in particular, via an interaction with the lipid scramblase XK. The latter localization seems to be most critical for disease. My talk will discuss our studies of this interaction and review our investigations of other VPS13 family members. I will also discuss major open questions concerning the function of this protein family.

**Sphingolipid and Phospholipid Levels Are Altered in Human Brain in Chorea-Acanthocytosis**

*Gabriel Miltenberger-Miltenyi*^1,2,3,4^*, Attila Jones*^5^*, Amber M. Tetlow*^6,7,8^*, Vasco A. Conceição*^4^*, John F. Crary*^6,7,8^*, Ricky Michael Ditzel Jr*.^6,7,8^*, Kurt Farrell*^6,7,8^*, Renu Nandakumar*^9^*, Brandon Barton*^10,11^*, Barbara I. Karp*^12^*, Alana Kirby*^11^*, Debra J. Lett*^13^*, Karin Mente*^14,15^*, Susan Morgello*^6,8,16^*, David K. Simon*^17,18^*, Ruth H. Walker*^16,19^

^1^ Laboratório de Genética, Faculdade de Medicina, Universidade de Lisboa, Lisbon, Portugal

^2^ Department of Neurology, Ludwig-Maximilians-Universität München, Munich, Germany

^3^ Reference Center on Lysosomal Storage Diseases, Hospital Senhora da Oliveira, Guimarães, Portugal

^4^ Instituto de Medicina Molecular João Lobo Antunes, Faculdade de Medicina da Universidade de Lisboa, Lisbon, Portugal

^5^ Laboratory of Behavioral Neuroscience, National Institute on Aging, National Institutes of Health, Baltimore, Maryland, USA

^6^ Department of Pathology, Molecular, and Cell Based Medicine, Icahn School of Medicine at Mount Sinai, New York City, New York, USA

^7^ Department of Neuroscience and Artificial Intelligence & Human Health, Icahn School of Medicine at Mount Sinai, New York City, New York, USA

^8^ Friedman Brain Institute, Ronald M. Loeb Center for Alzheimer’s Disease, Icahn School of Medicine at Mount Sinai, New York City, New York, USA

^9^ Biomarkers Core Laboratory, Irving Institute for Clinical and Translational Research, Columbia University Irving Medical Center, New York City, New York, USA

^10^ Rush University Medical Center, Chicago, Illinois, USA

^11^ Jesse Brown Veterans Affairs Medical Center, Chicago, Illinois, USA

^12^ Human Motor Control Section, National Institute of Neurological Disorders and Stroke, National Institutes of Health, Bethesda, Maryland, USA

^13^ Newcastle Brain Tissue Resource, Newcastle University, Newcastle, United Kingdom

^14^ Departments of Neurology and Pathology, Case Western Reserve University, Cleveland, Ohio, USA

^15^ Louis Stokes Cleveland VA Medical Center, Cleveland, Ohio, USA

^16^ Department of Neurology, Icahn School of Medicine at Mount Sinai, New York City, New York, USA

^17^ Beth Israel Deaconess Medical Center, Boston, Massachusetts, USA

^18^ Harvard Medical School, Boston, Massachusetts, USA

^19^ James J. Peters Veterans Affairs Medical Center, Bronx, New York, USA

**Background:** Chorea-acanthocytosis (ChAc) is associated with mutations of *VPS13A*, which encodes for chorein, a protein implicated in lipid transport at intracellular membrane contact sites.

**Objectives:** The goal of this study was to establish the lipidomic profile of patients with ChAc.

**Methods:** We analyzed 593 lipid species in the caudate nucleus (CN), putamen, and dorsolateral prefrontal cortex (DLPFC) from postmortem tissues of four patients with ChAc and six patients without ChAc.

**Results:** We found increased levels of bis(monoacylglycerol)phosphate, sulfatide, lysophosphatidylserine, and phosphatidylcholine ether in the CN and putamen, but not in the DLPFC, of patients with ChAc. Phosphatidylserine and monoacylglycerol were increased in the CN and N-acyl phosphatidylserine in the putamen. N-acyl serine was decreased in the CN and DLPFC, whereas lysophosphatidylinositol was decreased in the DLPFC.

**Conclusions:** We present the first evidence of altered sphingolipid and phospholipid levels in the brains of patients with ChAc. Our observations are congruent with recent findings in cellular and animal models, and implicate defects of lipid processing in VPS13A disease pathophysiology.

**The biology and regulation of bulk lipid transporters**

*Arash Bashirullah*

Pharmaceutical Sciences, School of Pharmacy, University of Wisconsin–Madison, USA

We helped define the Bridge-like Lipid Transfer Proteins (BLTPs), a novel superfamily that includes the neuroacanthocytosis-associated protein VPS13A, ATG2, and other less well-studied proteins. In addition to a signature hydrophobic core, we found that several of the BLTPs share a conserved lipid binding pocket. Importantly, patient-derived mutations map to this pocket, and these mutations disrupt proper targeting of BLTPs, specifically BLTP2, to membrane contact sites. We also identified a conserved adapter protein required for MCS targeting of BLTP2. Loss of MCS targeting results in cellular and physiological defects that are indistinguishable from loss-of-function mutations in BLTP2. Overall, these results highlight regulatory paradigms that are shared among BLTPs, demonstrating the utility of studying different members of this protein family and their interacting partners to uncover novel aspects of disease etiology.

## Bulk lipid transfer disorders – recent developments

**Recent advances in studies of VPS13A brain tissue**

*Ruth H. Walker, MB, ChB, PhD* ^1,2^

^1^ Department of Neurology, Icahn School of Medicine at Mount Sinai, New York, New York, USA

^2^ James J. Peters Veterans Affairs Medical Center, Bronx, NY, USA

Very little is known about the underlying pathophysiology of neurodegeneration in VPS13A and XK diseases. We are collecting donated brain tissues from patients with these disorders with the aim of systematically studying neuropathological, biochemical, and other aspects of brain changes due to mutations of both of the involved genes. To date we have collected brain tissue from seven patients with VPS13A disease from both US and international locations, and four patients with XK disease. Due to the historical nature of some of these donations, not all are appropriate for all proposed studies.

We have performed lipidomics studies of 3 brain regions from 4 patients with VPS13A disease, and, in comparison to 6 control brains, found significant differences in several specific lipid species. We performed neuropathological evaluation of brain tissue from 7 patients, including the 4 also studied by lipidomics. We found several features which had not been previously identified in VPS13A brain tissue; specifically calcifications of unusual pattern, occasional p62- and ubiquitin-immunoreactive cells, and intracellular inclusions of diverse morphologies which labelled with Luxol Fast Blue. In the one case in whom electron microscopy was possible (due to specific features of brain donation) we noted collections of electron-dense materials within neurons. We hypothesize that these features correspond to collections of lipid due to dysfunction of lipid metabolism, as reflected in our lipidomic studies. We also hypothesize that the other features noted, such as p62- and ubiquitin-immunoreactivity reflect dysfunctional autophagy, as has been found in model species such as *drosophila*. Parallel studies of XK disease brain tissues and tissues from other regions in both disorders are planned.

**High-content drug screening for Cohen Syndrome**

*Fabrizio Vacca* ^1,2^ *, Romain Da Costa* ^1^ *, Ruth Choung* ^1^ *, Huda Barakullah* ^1^ *, Howard Riezman* ^2^ *, Muhammad Ansar* ^1,3^

^1^ Department of Ophthalmology, University of Lausanne, Jules Gonin Eye Hospital, Fondation Asile Des Aveugles, Lausanne, Switzerland

^2^ Dept. of Biochemistry, University of Geneva, Geneva, Switzerland

^3^ Advanced Molecular Genetics and Genomics Disease Research and Treatment Centre, Dow University of Health Sciences, Karachi, Pakistan

Cohen Syndrome (CS) is a rare autosomal recessive disorder caused by biallelic loss-of-function mutations in the *VPS13B* gene. It presents with multiple clinical features, including microcephaly, developmental delay, intellectual disability, neutropenia, and retinal degeneration. VPS13B is part of the chorein-domain protein family, characterized by a long hydrophobic groove, proposed to mediate lipid transfer between membranes. Precise VPS13B function and lipid selectivity are not yet established, but the fragmentation of the Golgi complex is the more evident hallmark of VPS13B deficient cells. We used morphological defects of the Golgi complex to develop a microscopy-based assay for high-throughput screening of small molecules.

We screened the Prestwick chemical library, composed of 1280, mostly FDA-approved, compounds. We identified two different classes of compounds that efficiently recovered Golgi morphology in VPS13B knockout Hela cells: glucocorticoid receptor agonists and lysosomotropic drugs, which act as functional inhibitors of acidic lipases. The effect of lysosomotrophic drugs seems mediated by lysosomal storage, since the correction of Golgi morphology is mimicked by silencing the Niemann-Pick C1 gene, associated with a lysosomal storage disorder. Results were further confirmed in patient-derived fibroblasts for a selected number of candidate drugs.

We performed lipidomic analysis and found a general reduction in sphingolipids in VPS13B KO cells. Among sphingolipids, a more striking decrease was observed in C18 n-acyl species of sphingomyelin (SM). These SM species, while low abundant, are strongly enriched in COPI vesicles and are proposed to play a crucial role in Golgi to ER transport, linking this observation with the Golgi phenotype. Interestingly, all tested drugs were able to recover the cellular level of C18 SM species in VPS13B KO cells, indicating a potential mechanism of action of this class of drugs.

In conclusion, we developed a robust cell-based assay for screening small molecules and identified potential drug candidates for CS. Candidate drugs will be further tested in human brain organoids and in the CS mouse model.

**Using human cells to validate drugs effective in a yeast model of chorea acanthocytosis**

*Joanna Kamińska* ^1^ *, Dagmara Kabzińska* ^2^ *, Andrzej Kochański* ^2^ *, Teresa Żołądek* ^1^

^1^ Institute of Biochemistry and Biophysics Polish Academy of Sciences, Poland

^2^ Neuromuscular Unit, Mossakowski Medical Research Institute Polish Academy of Sciences, Poland

Mutations in the *VPS13A* gene, which result in chorea acanthocytosis, cause a number of changes in cells at the molecular level. VPS13A is a lipid transporter, thus, the main defect is in lipid transfer between organelles at membrane contact sites, but this results in the appearance of numerous molecular phenotypes. It is not known which of the phenotypes are primary, resulting directly from the lack of lipid transfer, and which are secondary, compensating for the primary defect. Although it is not known how molecular changes reflect the real disease pathology, it is important to develop therapies that target the molecular basis and reduce various changes at the cellular level. To achieve this goal, we have screened the Prestwick Chemical Library of FDA-approved drugs and a custom library of natural compounds using a previously established *vps13*Δ yeast model, and identified several active compounds (reviewed in Kaminska *et al.*, 2023, DOI: 10.3390/ijms23095106). Now we are using HeLa human cells in which the *VPS13A* gene has been silenced by siRNA technique to validate these compounds. This model is suitable, as in most cases mutations in the *VPS13A* gene result in the absence of the VPS13A protein. Several phenotypes described in the literature, including defect of autophagy and the activity of several kinases, were analysed in these cells and used for testing the first group of compounds, flavonoids, which contain luteolin. These compounds have the potential to affect at the molecular level the changes relevant to disease progression.

**The wide neuromuscular spectrum of VPS13A disease demonstrated by a case series**

*Anne Buchberger* ^1^ *, Kevin Peikert* ^2,3,4^ *, Alexander Mensch* ^5^ *, Tobias Haack* ^6,7,8^ *, Jan Kirschke* ^9^ *, Holger Prokisch* ^8^ *, Andreas Hermann* ^2,3,10^ *, Christian Mawrin* ^11^ *, Benedikt Schoser* ^12^ *, Adrian Danek* ^13^ *, Marcus Deschauer* ^1^ *, Isabell Cordts ^1^ *

^1^ Department of Neurology, Klinikum rechts der Isar, Technical University of Munich, Munich, Germany

^2^ Translational Neurodegeneration Section “Albrecht Kossel”, Department of Neurology, University Medical Center Rostock, University of Rostock, Rostock, Germany

^3^ Center for Transdisciplinary Neurosciences Rostock (CTNR), University Medical Center Rostock, University of Rostock, Rostock, Germany

^4^ United Neuroscience Campus (UNC) Lund – Rostock, Rostock, Deutschland

^5^ Department of Neurology, Martin Luther University Halle-Wittenberg and University Hospital Halle, Halle (Saale), Germany

^6^ Institute of Medical Genetics and Applied Genomics, University of Tübingen, Tübingen, Germany

^7^ Center for Rare Diseases, University of Tübingen, Tübingen, Germany

^8^ Institute of Human Genetics, Technical University of Munich, Munich, Germany

^9^ Department of Neuroradiology, Klinikum rechts der Isar, Technical University of Munich, Munich, Germany

^10^ German Center for Neurodegenerative Diseases (Deutsches Zentrum für Neurodegenerative Erkrankungen – DZNE), Rostock/Greifswald, Rostock, Germany

^11^ Department of Neuropathology, University of Magdeburg, Magdeburg, Germany

^12^ Friedrich-Baur-Institute, LMU Clinics, Department of Neurology, Ludwig Maximilian University of Munich, Munich, Germany

^13^ Department of Neurology, LMU Clinics, Ludwig Maximilian University of Munich, Munich, Germany

* both authors contributed equally

VPS13A disease is a rare, autosomal recessive neurodegenerative disorder that is characterized by a spectrum of movement disorders (chorea, dystonia, tics, sometimes parkinsonism), seizures, as well as psychiatric, cognitive, and behavioral changes. Neuromuscular involvement may include areflexia and muscle weakness and can often be rather mild, while creatine kinase is commonly elevated. Here, we present four cases of VPS13A disease with varying severity of neuromuscular symptoms. In all cases, bi-allelic *VPS13A* variants were confirmed. Age of onset ranged from 14 to 38 years, first sign being seizures in two patients and hyperkinesia and muscle weakness each in one patient. At presentation, areflexia was evident in all but one case. All patients showed pallhypesthesia. Two cases were mostly subclinically affected. In contrast, severe muscle weakness was present in two patients, leading to loss of walking ability due to progressive atrophic paresis of the proximal lower and distal upper limb muscles in one case. Elevated creatine kinase (max.: 1643 U/l; 2490 U/l; 2817 U/l; 5100 U/l) and acanthocytes were detected in all patients. Chorein in Western Blot from red blood cells was reduced in one patient and negative in three. Nerve conduction studies showed a predominantly axonal sensory to sensorimotor neuropathy in three out of four cases. Electromyography revealed neurogenic changes in all four patients and additional myopathic patterns in one case. Dixon MRI disclosed proximally accentuated oedema in all four cases (most prominent in both patients with clinically apparent muscle involvement), as well as fatty atrophy of the leg and trunk muscles in the two cases with muscular weakness. Biceps and quadriceps femoris muscles were most frequently impaired, showing oedema or atrophy in four and three patients respectively. Myohistological examination of the vastus lateralis muscle, currently only available for one individual with the most prominent muscular phenotype, revealed both neurogenic patterns with grouping of highly atrophic fibers and myopathic changes with fiber splitting and internalized nuclei. Electron microscopy showed structurally normal mitochondria, but multiple atypical electron dense lipid deposits and partially distended endoplasmic reticulum.

This case series demonstrates the wide neuromuscular spectrum of VPS13A disease, which, especially with regard to severe muscular involvement, has previously been underrecognized. A thorough description of the phenotypic spectrum supports both early diagnosis for future patients and comprehension of underlying pathophysiological mechanisms.

## Disease insights from cell models

**Calcium phenotype in iPSC-derived neurons from patients with VPS13A disease**

*Dajana Großmann* ^1^ *, Emily Fischer* ^1^ *, Sebastian Klicker* ^1^ *, Kevin Peikert* ^1,2,3^ *, Andreas Hermann* ^1,2,4^

^1^ Translational Neurodegeneration Section “Albrecht-Kossel”, Department of Neurology, Rostock University Medical Center, University of Rostock, Germany

^2^ Center for Transdisciplinary Neurosciences Rostock (CTNR), University Medical Center Rostock, Rostock, Germany

^3^ United Neuroscience Campus Lund-Rostock (UNC), Rostock site

^4^ Deutsches Zentrum für Neurodegenerative Erkrankungen (DZNE) Rostock/Greifswald, Rostock, Germany

**Background:** Membrane contact sites (MCS) are critically involved in the function and maintenance of several organelles, including mitochondria and the endoplasmic reticulum (ER). MCS are major hubs for the exchange of metabolites, ions, proteins or lipids. VPS13A is a membrane-residing protein bridging two membranes to enable bulk lipid transfer between organelles. Loss of function mutations in the *VPS13A* gene cause VPS13A disease/ chorea-acanthocytosis, a neurodegenerative disorder of the young adulthood. Previous studies showed alterations of lipid transfer and disruption of calcium homeostasis due to VPS13A deficiency.

**Objective:** Although MCSs are becoming increasingly important in neurodegenerative disease research, their contribution to neuronal death is still largely unclear. We attempt to investigate the function of MCSs to better understand the consequences of their dysfunction in the context of VPS13A disease.

**Methods:** We use iPSC-derived neurons from patients harbouring mutations in *VPS13A*, leading to loss of VPS13A protein. Neurons are transfected with a specific plasmid-based marker for mitochondria-ER contact sites (MERCS). Twenty-four hours post-transfection neurons were labelled with fluorescent probes staining mitochondria and calcium. We use a LSM900 confocal microscope with Airyscan 2 module (Zeiss) for live-cell super resolution imaging in order to assess calcium transfer at MERCS.

**Results:** We observed a mitochondrial calcium overload in VPS13A-deficient neurons. In depth analysis on calcium transfer and its regulation at mitochondria are currently performed.

**Conclusion:** Our results support the critical involvement of VPS13A in calcium homeostasis at mitochondria. Further research is needed to improve our understanding of the effects of calcium transfer between organelles on neuronal function and demise.

**Corticostriatal knockdown of VPS13A disrupts locomotion and synaptic plasticity through mitochondrial function impairment**

*Esther García-García* ^1,2,3^ *, Alba Ramón-Lainez* ^1,2,3^ *, Sara Conde-Berriozabal* ^1,2,3^ *, Mercè Masana* ^1,2,3^ *, Jordi Alberch* ^1,2,3^ *, Manuel J. Rodríguez* ^1,2,3^

^1^ Department of Biomedical Sciences, Institute of Neurosciences, School of Medicine and Health Sciences, Universitat de Barcelona, Spain

^2^ Institut d’Investigacions Biomèdiques August Pi i Sunyer (IDIBAPS), Barcelona, Spain

^3^ Centro de Investigación Biomédica en Red de Enfermedades Neurodegenerativas (CIBERNED), Madrid, Spain

Chorea-acanthocytosis (ChAc) is an inherited neurodegenerative movement disorder caused by VPS13A gene mutations leading to the absence of protein expression. The striatum is the most affected brain region in ChAc patients. However, the study of the VPS13A function in the basal ganglia has been poorly addressed. We analyzed the involvement of VPS13A in basal ganglia synaptic plasticity and behavior and then studied the effects of VPS13A knockdown (KD) in neuronal ER-mitochondria contact homeostasis. VPS13A-KD neurons in vitro showed molecular synaptic alterations. In the animal model, VPS13A-KD mice showed modification of the locomotor behavior pattern, with increased exploratory behavior and hyperlocomotion. Corticostriatal dysfunction in VPS13A-KD mice was evidenced by impaired striatal LTD after stimulation of cortical afferents, which was partially recovered by BDNF administration. VPS13A-KD did not lead to neuronal loss in the cortex or the striatum but induced a decrease in the neuronal release of CX3CL1 and triggered a microglial reaction, especially in the striatum. Notably, CX3CL1 administration also partially restored the impaired corticostriatal LTD in VPS13A-KD mice. We next analyzed in vitro the effects of VPS13A KD on mitochondria homeostasis of cortical neurons. We also explored in vivo the VPS13A cellular interactions by means of specific protein immunoprecipitation followed by mass spectrometry. In cortical neurons, we found VPS13A located in neuronal MAMs and that VPS13A-KD specifically alters mitochondrial homeostasis. In the mouse brain, VPS13A specifically interacts with enzymes of the tricarboxylic acid cycle and with proteins involved in mitochondrial DNA repair. These interactions reinforce the role of VPS13A in the maintenance of mitochondrial homeostasis and unveil its involvement in the regulation of neuronal mitochondrial activity and energy metabolism with important implications for neuronal plasticity.

To sum up, we report that VPS13A plays a key role in the modulation of neuronal communication and synaptic plasticity in the basal ganglia. Particularly VPS13A-KD induces mitochondrial dysfunction and impairs corticostriatal synaptic plasticity, suggesting that a disruption of these processes may account for the early pathophysiological stages of ChAc. Learning more about BDNF-mediated synaptic plasticity and the contribution of mitochondrial homeostasis to synaptic plasticity could give us more insight into ChAc pathophysiology, allowing for the exploration of new therapeutic approaches.

**Disease relevance of rare VPS13B missense variants for Cohen syndrome**

*Wenke Seifert*

Institute of Cell Biology and Neurobiology, Charité – Universitätsmedizin Berlin – CCM, Berlin, Germany

The autosomal recessive inherited Cohen syndrome is a neurodevelopmental disorder majorly characterized by postnatal microcephaly, intellectual disability, and a typical facial gestalt. Genetic variants in *VPS13B* have been identified to cause Cohen syndrome, but have been also linked to autism, retinal diseases, primary immunodeficiency, and short stature. While it is well established that loss-of-function mutations in *VPS13B* cause Cohen syndrome, the contribution of missense variants to the pathomechanism remains unexplained. The investigation of *VPS13B* missense variants is therefore crucial for the classification of their disease relevance. The interpretation of *VPS13B* missense variants is difficult due to a lack of detailed VPS13B protein function and a clear domain structure. *VPS13B* encodes one of four mammalian pioneer VPS13 family proteins. VPS13B protein (450 kDa) is membrane-associated and critically regulates the integrity of the Golgi complex. Our study on previously published disease-associated *VPS13B* missense variants explores their pathogenic impact by a systematic reassessment of clinical patient information, comprehensive in silico predictions, and initial functional investigation. In vitro analysis of 10 subcloned *VPS13B* missense variants resulted in full-length proteins after transient overexpression. 6 out of these 10 *VPS13B* missense variants show a decreased enrichment at the Golgi complex in steady state. Accordingly, overexpression of these *VPS13B* missense variants did not rescue Golgi fragmentation following RNAi-mediated depletion of endogenous VPS13B. Together with the clinical and in silico data our experiments validate 6/10 variants as likely pathogenic according to American College of Medical Genetics classification. It is noteworthy that 3 likely pathogenic variants of *VPS13B* group to the specific protein domain (SHR_BD), which suggests an influence on phosphatidylinositol-3-phosphate binding. Our study underlines that the interpretation of *VPS13B* genetic variants is crucially determined by the integration of clinical, genetic, in silico, and experimental data in order to define more precise pathomechanisms of genetically inherited neuronal diseases.

## Disease insights from model organisms

**Suppressor mutations in yeast VPS13 suggest a cooperative mechanism for lipid transport**

*Aaron Neiman and Jae-Sook Park*

Department of Biochemistry and Cell Biology, Stony Brook University, NY, USA

Yeast cells lacking the ER-mitochondria encounter site (ERMES) complex cannot grow in the absence of glucose due to mitochondrial defects. However, spontaneous suppressors arise that allow growth on non-fermentable carbon sources. These suppressors are caused by point mutations in the yeast *VPS13* gene that, presumably, enhance lipid transfer to the mitochondrion and compensate for the loss of ERMES. Earlier studies suggested that suppression was due to altered intracellular distribution of VPS13 that increased localization of the mutant protein to mitochondrial membrane contact sites. However, we have found that the same mutation that bypasses the need for ERMES can also partially compensate for defects in VPS13 function at the endosome and at the prospore membrane, which cannot be explained by increased occupancy at mitochondrial contacts. Rather, these results suggest that these mutations make the mutant VPS13 more efficient at lipid transfer.

More than a dozen different suppressor alleles of *VPS13* have been isolated. These mutations are spread out over a 1500 amino acid region of the protein comprising most of the RBG repeats that make up the hydrophobic channel. Mapping these mutations onto the Alphafold structure reveals that they can occur in at least seven different RBG repeats (of 12 total) and that they are enriched at positions in the hinge region of the repeat. There is little primary sequence identity between RBG repeats. However, using a structural alignment of different RBG domains allowed us to identify the residue in a given RBG repeat that corresponds to the position of a suppressor mutation in another RBG repeat. This revealed that several suppressors are located at the same position in different repeats. Moreover, we were able to engineer new suppressor mutations using these alignments. That mutations in a (possibly) flexible hinge region of the RBG repeat gives rise to alleles with enhanced lipid transfer activity suggesting that the mechanism of transfer involves a conformational change in the RBG repeat. That single mutations in different RBG repeats produce the same phenotype further suggests that this conformational change involves cooperative movement of the repeats. A model incorporating these observations will be discussed.

**Molecular mechanisms underlying Chorea Acanthocytosis**

*Genesis Rodriguez* ^1,2^ *, Elizabeth Tank* ^2^ *, Roberta Fuller* ^1,3^ *, Andrew Tidball* ^2^ *, Tracy Qiao* ^2^ *, Jack Parent* ^1,2^ *, Sami Barmada* ^1,2^

^1^ Neuroscience Graduate Program, University of Michigan, Ann Arbor, MI, USA

^2^ Department of Neurology, University of Michigan, Ann Arbor, MI, USA

^3^ Department of Biological Chemistry, University of Michigan, Ann Arbor, MI, USA

Chorea acanthocytosis (ChAc) is an autosomal-recessive neurodegenerative disorder caused by mutations in the gene encoding vacuolar protein sorting factor 13A (VPS13A). Structural and functional studies show that VPS13A aids in the transport of lipids between organelles such as the ER and mitochondria. Together with the fact that the VPS13A N-terminal region is homologous to the evolutionarily conserved autophagy-related genes (*Atg*) protein family, these data suggest a function for VPS13A in specialized branches of autophagy, namely ER-phagy and mitophagy. Using HEK293T cells in which the native *VPS13A* has been knocked out (VPS13A KO) via CRISPR, we found that VPS13A loss only moderately affects the levels of receptors involved ER-phagy and mitophagy, and autophagic flux was only mildly affected by VPS13A KO. Furthermore, mass spectroscopy and unbiased proteomics failed to demonstrate an upregulation of ER-phagy and mitophagy substrates in VPS13A KO HEK293T cells, arguing against an essential role for VPS13A in maintaining these processes. Therefore, we broadened our approach and investigated candidate proteins that were significantly overrepresented VPS13A KO HEK293T cells. Candidates that were consistently affected at the protein level also demonstrated changes at the RNA level, implying transcriptional regulation upon VPS13A KO. To investigate whether VPS13A may directly or indirectly regulate transcription, we conducted next generation RNA-sequencing in VPS13A KO HEK293T cells and controls. These findings imply that VPS13A may be regulating RNA expression directly or indirectly, through secondary effects on mRNA transport or transcription factors. Concurrent with these studies, we knocked out VPS13A from human induced pluripotent stem cells (iPSCs) taken from healthy donors. These VPS13A KO cells displayed an elevated risk of death on longitudinal analyses, similar to what we observed in iPSC-derived neurons from ChAc patients and rodent primary cortical neurons in which VPS13A has been deleted by CRISPR. Future studies will examine cell type-specific functions of VPS13A in human neurons compared to HEK293T cells, with a focus on pathways that mediate neuronal survival. Collectively, these investigations will help define a function for VPS13A in neurons, outline disease mechanisms, and highlight pathways that may be targeted to prevent neuron loss in ChAc.

**Funding:** The Weinbaum Lakritz Research Fund

**Genetic and physical interaction of VPS13 lipid transporter with Rsp5 E3 ubiquitin ligase: coordination of lipid synthesis with lipid transport?**

*Joanna Kamińska* ^1^ *, Weronika Rzepnikowska* ^2^ *, Teresa Żołądek* ^1^

^1^ Institute of Biochemistry and Biophysics, Polish Academy of Sciences, Warsaw, Poland

^2^ Neuromuscular Unit, Mossakowski Medical Research Institute, Polish Academy of Sciences, Warsaw, Poland

Several rare neurodegenerative diseases depend on mutations in *VPS13A-D* genes, including chorea-acanthocytosis and early-onset Parkinsonism. *VPS13* genes are conserved from yeast to humans and yeast with unique *VPS13* is a good model system to study function of VPS13 proteins. Recent findings show that VPS13 protein transports bulk lipids at membrane contact sites. Localization of VPS13 is dependent on interactions with specific proteins and lipids which are characteristic to different subcellular compartments. The lipids determining the localization of VPS13 are quite well defined, but the proteins are not. To investigate VPS13 protein partners, we purified VPS13 from yeast cells by pull down and identified interacting proteins using mass spectrometry. Among proteins recognized, we found RSP5 ubiquitin ligase. The VPS13-RSP5 interaction was confirmed by immunoprecipitation and Western Blot. RSP5 is important for the lipid homeostasis. It ubiquitinates and is involved in proteasomal maturation of the transcriptional activators, SPT23 and MGA2, which regulate the expression of genes encoding lipid biosynthetic enzymes. Increased levels of activated SPT23 or MGA2 result in the formation of gigantic lipid droplets. This prompted us to analyse genetic interaction between VPS13 and RSP5 genes and we found that additional copies of the RSP5 gene introduced into VPS13Δ mutant cells increase their sensitivity to sodium dodecyl sulphate. We also observed the lower level of Rsp5 E3 ubiquitin ligase in VPS13Δ compared to the wild type cells. These results suggest that the VPS13-RSP5 interaction may be important for coordination of the lipid synthesis with lipid transport capacity of the cell.

**In Vps13a**^–/–^ **mice, the impairment of autophagy exacerbates skeletal muscle aging phenotype**

*Veronica Riccardi* ^1^ *, Enrica Federti* ^1^ *, Carlo Viscomi* ^2^ *, Marco Sandri* ^3^ *, Alessandro Matte* ^1^ *, Immacolata Andolfo* ^4,5^ *, Federica Gevi* ^6^ *, Alice Passarini* ^1^ *, Leonardo Salviati* ^7^ *, Anna Maria Timperio* ^8^ *, Alexander Mensch* ^9^ *, Marcus Deschauer* ^10^ *, Kevin Peikert* ^11,12^ *, Seth L Alper* ^13^ *, Achille Iolascon* ^4,5^ *, Adrian Danek* ^14^ *, Ruth H. Walker* ^15^ *, Andreas Hermann* ^11,12,16,17^ *, Lucia De Franceschi* ^1^

^1^ Department of Medicine, University of Verona, Policlinico GB Rossi, P.Le L. Scuro, 10, 37134, Verona, Italy

^2^ Department of Bomedical Sciences, University of Padova, Via Ugo Bassi 58b, 35128 Padova, Italy

^3^ Venetian Institute of Molecular Medicine, 35129 Padova, Italy; Department of Biomedical Sciences, University of Padova, 35100 Padova, Italy

^4^ Dipartimento di Medicina Molecolare e Biotecnologie Mediche, Università degli Studi di Napoli Federico II, Napoli, Italy

^5^ CEINGE Biotecnologie Avanzate, Napoli, Italy

^6^ Department of Biology and Ecology, University of Tuscia, 01100 Viterbo, Italy

^7^ Clinical Genetics Unit, Department of Women and Children’s Health, University of Padova, Padua, Italy

^8^ Department of Biology and Ecology, University of Tuscia, 01100 Viterbo, Italy

^9^ Department of Neurology, Martin Luther University Halle-Wittenberg and University Hospital Halle, Halle (Saale), Germany

^10^ Department of Neurology, School of Medicine, Technical University Munich, Munich, Germany

^11^ Translational Neurodegeneration Section “Albrecht-Kossel”, Department of Neurology, University Medical Center Rostock, University of Rostock, Gehlsheimer Straße 20, 18147, Rostock, Germany

^12^ Division for Neurodegenerative Diseases, Department of Neurology, Technische Universität Dresden, Dresden, Germany

^13^ Division of Nephrology and Vascular Biology Research Center, Beth Israel Deaconess Medical Center and Department of Medicine, Harvard Medical School, Boston, MA, USA

^14^ Neurologische Klinik und Poliklinik, Ludwig-Maximilians-Universität, Munich, Germany

^15^ Department of Neurology, James J. Peters Veterans Affairs Medical Center and Mount Sinai School of Medicine, Bronx, New York, USA

^16^ Center for Transdisciplinary Neurosciences Rostock (CTNR), University Medical Center Rostock, University of Rostock, Rostock, Germany

^17^ Center for Regenerative Therapies Dresden, Dresden, Germany

Chorea-acanthocytosis (ChAc)/VPS13A disease is an ultra-rare, highly invalidating disease due to mutations in the *VPS13A* gene, encoding for chorein. One of the early signs of ChAc is an increased serum creatine kinase (CK) of muscular origin, in some cases associated with progressive muscular weakness. Here, we analyzed skeletal muscles from mice genetically lacking *Vps13a* (Vps13a^–/–^), a mouse model of ChAc.

To avoid any aging effect on skeletal muscle, we focused on 8 months old mice. Vps13a^–/–^ mice displayed higher plasma CK than gender and age matched wild-type animals. This was associated with sarcopenia. Metabolomic analysis revealed alterations in protein and lipid muscle cell oxidation and abnormalities in tryptophan and catecholamine pathways with accumulation of serotonin, suggesting a perturbation of protein trafficking in neuromuscular junction associated with oxidation. Since autophagy plays an important role in muscle cell homeostasis, we evaluated key proteins of autophagy in muscles from Vps13a^–/–^ mice. We found increased LC3-II, associated with accumulation of autophagy related proteins such as Atg5, 7, Rab 3, Lamp1 and p62, suggesting an impairment of autophagy. This was further corroborated by the lack in the activation of autophagy in response to starvation or to starvation plus colchicine. In agreement, we found accumulation of active Lyn and of polyubiquinated K48 proteins. This promotes ER stress and activation of the unfolded protein response systems in Vps13a^–/–^ mice muscles. The pro-oxidant environment characterizing muscles from Vps13a^–/–^ mice promotes the activation of NF-kB and Nrf2, two important transcriptional factors involved in cell response against oxidation and inflammation. Indeed, we observed local up-regulation of gene expression of cytokines such as *Il1b* and *TNF*α as well as Nrf2-related antioxidant systems.

Taken together, our data suggest a possible acceleration of muscle senescence as previously reported in a mouse model genetically lacking *Atg7* (Sandri M et al Cell Reports 8: 1509, 2014). Indeed, we found accumulation of NCAM-1, neuronal cell-adhesion molecule-1, which is a glycoprotein expressed in muscular junction and used as marker of myofiber denervation. In muscles from Vps13a^–/–^ mice, the amount of NCAM-1 was higher than in muscles from wild-type animals. Finally, we took advantage of the availability of muscle biopsies from patients with ChAc. In human samples, we found accumulation of autophagy related proteins in presence of increased LC3-II, similarly to that observed in Vps13a^–/–^ mice. This was associated again with increased protein oxidation and activation of NF-kB transcriptional factor, paralleling the findings in the murine model for ChAc. Noteworthy, accumulation of NCAM-1 was also observed in muscle biopsies from ChAc patients when compared to healthy controls.

In conclusion, our data identify a metabolic perturbation of muscles from Vps13a^–/–^ mice, suggesting accelerated aging. In conclusion, in skeletal muscle the absence of chorein promotes an impairment of autophagy associated with accumulation of poly-ubiquitinated proteins and ER stress. This exacerbates muscle aging phenotype, as supported by the accumulation of NCAM-1. Collectively, our data identify a novel mechanism responsible for muscle sufferance in ChAc, highlighting the link between the absence of chorein, impaired autophagy and early aging muscle phenotype.

## “Glenn Irvine Prize” lecture

**A translational perspective on neuroacanthocytosis syndromes: from drug target identification to potential treatment strategies, from natural history to clinical trial readiness**

*Kevin Peikert*

Translational Neurodegeneration Section “Albrecht Kossel”, Department of Neurology, University Medical Center Rostock, University of Rostock, Rostock, Germany

Center for Transdisciplinary Neurosciences Rostock (CTNR), University Medical Center Rostock, University of Rostock, Rostock, Germany

United Neuroscience Campus (UNC) Lund – Rostock, Rostock, Germany

Thanks to remarkable scientific advances in our field in recent years, the pathophysiological understanding of the core neuroacanthocytosis syndromes, VPS13A and XK disease, has become much clearer: First, the interaction of the underlying proteins, VPS13A and XK, in a complex helps us to better understand the phenotypical similarities of these diseases. Second, their function as bridge-like lipid transfer protein and scramblase, respectively, seem to turn the conditions (together with related diseases) central for a new pathophysiological paradigm: disorders of bulk lipid transfer.

However, VPS13A and XK disease are still incurable, progressive neurodegenerative disorders. Our collaborative work therefore focused on identifying potential drug targets, such as Lyn kinase, for VPS13A disease, and on an early “bench-to-bedside” translation of these findings. In this context, limited knowledge on the natural history and the lack of appropriate biomarkers became obvious major barriers for “clinical trial readiness”.

In this talk, I will summarize our efforts on drug target identification and “lessons learned” from the first disease-modifying treatment approaches in VPS13A disease. Progress toward clinical trial readiness implies the establishment of novel biomarkers, disease phenotyping, case identification, and the need for international cohort building.

## Red blood cell-related research

**Red blood cells in the Neuroacanthocytosis syndromes**

*Lars Kaestner*

Theoretical Medicine and Biosciences, Saarland University, Campus University Hospital, Homburg/Saar Germany

Dynamics of Fluids, Experimental Physics, Saarland University, Saarbruecken, Germany

The red blood cell shape gives the Neuroacanthocytosis syndromes the name – at least partly. Contrary, the acanthocytes make only a small portion of the red blood cells and this portion is variable from patient to patient. In the past, it has been difficult to reproducibly quantify the portion of acanthocytes but this could be solved by confocal 3D-imaging of glutaraldehyde-fixed red blood cells and shape classification based on artificial neural networks.

Whether the red blood cell properties contribute to disease symptoms and/or disease progression is still elusive. The occurrence of neural complications in other diseases with misshapen red blood cells such as sickle cell disease or thalassemia suggest a causal relation. However, the slow disease progression of the Neuroacanthocytosis syndromes makes investigations and proof difficult.

Independent of a putative causal nature of red blood cells in the Neuroacanthocytosis syndromes, the biophysical properties of the acanthocytes (as well as the other red blood cells from Neuroacanthocytosis syndrome patients) provide the potential of being biomarkers. Examples of such red blood cell-derived biophysical properties are the erythrocyte sedimentation rate, which is significantly prolonged in the blood of Neuroacanthocytosis syndrome patients, or the shape analysis of red blood cells in microfluidic capillary flow.

**Evaluation of red blood cell membrane lipid and spectrin distribution in Chorea-acanthocytosis and McLeod syndrome**

*Marine Ghodsi* ^1^ *, Anne-Sophie Cloos* ^1^ *, Amaury Stommen* ^1^ *, Andreas Hermann* ^2,3,4^ *, Kevin Peikert* ^2,3,5^ *, Donatienne Tyteca* ^1^

^1^ CELL Unit and PICT Platform, de Duve Institute, Brussels, Belgium

^2^ Translational Neurodegeneration Section “Albrecht Kossel”, Department of Neurology, University Medical Center Rostock, University of Rostock, Germany

^3^ Center for Transdisciplinary Neurosciences Rostock (CTNR), University Medical Center Rostock, Rostock, Germany

^4^ Deutsches Zentrum für Neurodegenerative Erkrankungen (DZNE) Rostock/Greifswald, Rostock, Germany

^5^ United Neuroscience Campus Lund-Rostock (UNC), Rostock site

Chorea-acanthocytosis and McLeod syndrome are due to mutations in *VPS13A* and *XK*, respectively, but share similar clinical manifestations: movement disorders due to degeneration of the caudate nucleus and acanthocytes, i.e. spiculated red blood cells (RBCs). Both VPS13A and XK are thought to control bilayer lipids dynamics, through net lipid transfer between adjacent cytosolic membrane leaflets (e.g. the endoplasmic reticulum, mitochondria and lipid droplets) and lipid scrambling to equilibrate lipids between bilayer leaflets, respectively. We here determined the consequences of those mutations for RBC morphology, membrane lipid transversal asymmetry (phosphatidylserine surface exposure), membrane lipid lateral distribution in domains (abundance of cholesterol- and sphingomyelin-enriched domains per hemi-RBC area), spectrin cytoskeleton (spectrin membrane occupancy and heterogeneous distribution in patches) and RBC maturation (RBCs with ceramide-enriched patches) of 5 patients suffering from Chorea-acanthocytosis and 2 patients with McLeod syndrome compared to gender- and age-matching healthy control subjects. Our data revealed that none of the patients were altered for phosphatidylserine surface exposure, suggesting preservation of transversal membrane asymmetry. In contrast, all of them showed increased abundance of acanthocytes and impaired abundance and distribution of cholesterol- and sphingomyelin-enriched domains, but to a differential extent. Those parameters well correlated. Thus, the higher the acanthocyte number, the higher the abundance of sphingomyelin-enriched domains and the lower the abundance of cholesterol-enriched domains. Moreover, patients showing the highest number of cholesterol-enriched domains also exhibited higher number of RBCs with ceramide and spectrin-enriched patches, suggesting RBC maturation defects. Altogether, our data indicate that acanthocytes are associated with membrane lipid lateral asymmetry and cytoskeleton defects. Furthermore, RBC precursors might also be affected in some patients, leading to disturbed RBC maturation. Further studies are needed to unravel the mechanism behind cytoskeletal and membrane alterations and their potential relationship with lipid content changes.

**Red blood cells: how they are born, how do they live, and how they die happily thereafter**

*Giampaolo Minetti*

Department of Biology and Biotechnology “L. Spallanzani”, University of Pavia, Pavia, Italy

As non-nucleated cells, mammalian red blood cells (RBCs) cannot replicate, and yet they must be able to survive in the circulation for a relatively long time. They rely on intrinsic mechanisms for energy supply (ATP and reducing power) to sustain ion homeostasis, redox balance and limited repair mechanisms. On the other hand, as specialization implies loss of autonomy, they undergo continuous preventive maintenance by systemic quality control mechanisms that involve multiple organs and tissues (spleen, liver, endothelium). Circulating RBCs are commonly seen as a single cell population, although they differ by age. They originate from reticulocytes (Retics) that are produced in the bone marrow by spontaneous enucleation of orthochromatic erythroblasts, and undergo an initial maturational phase while still in the marrow through complex but fairly well characterized processes of autophagy, proteolysis, vesicular trafficking, and controlled volume decrease, to reduce cell size and remove unneeded components. However, by the time Retics exits the marrow niche, they must still complete maturation, through additional loss of surface area and volume and of residual constituents in the membrane and in the cytoplasm (transferrin receptor -TfR-, ribosomal RNA). Most importantly, the cell must attain mechanical stability through the complete assembly of the membrane skeleton and its anchoring to the lipid bilayer. The mechanism(s) by which Retics complete maturation in the circulation are still unknown, but most likely differ from those at play in the marrow phase of maturation, where the complex molecular machinery becomes progressively exhausted. In particular, any major remodelling of the plasma membrane can no longer be carried out spontaneously by the cell, because eukaryotic cells rely on complex vesicular trafficking with internal membranes for this purpose, and internal membranes are no longer present in circulating Retics. We have recently described that even the complete absence of TfR and RNA is not sufficient to classify the cell as a mature RBC. Young RBCs are, from the point of view of the membrane lipid composition and mechanical stability, more similar to Retics than to mature RBCs. The striking remodelling of the lipid component in the membrane, which must also involve active “membrane conditioning” by external players (macrophages, endothelial cells) produces a net decrease in surface area whereby some phospholipids become enriched and some depleted, by a complex process of lipid exchange with plasma lipoproteins, reacylation of individual phospholipids in the cell and lipid translocation across the bilayer (scramblases, flippases floppases). The net decrease in membrane area must be accompanied by a parallel, coordinated reduction in cell volume, by engagement of channels that allow the efflux of K+, Cl- and osmotic water. Remarkably, because the relative abundance of sphingomyelin and cholesterol increases as a result of the lipid remodelling, a higher liquid ordered state of membrane lipids is generated (cholesterol and sphingolipids are enriched in membrane rafts). It is the combined action of lipid remodelling and the reduction in cell size that drives the completion of membrane skeleton assembly and the attainment of the condition of a stable, mature RBC. Therefore, although it was previously believed that Retics take 24–48 h to become mature RBCs in the circulation, the mature state is not reached before one week from the time the Retic enters the circulation. Mature RBCs circulate as stable and efficient cells for their entire life span, without major alterations of their metabolic capacity, lipid composition, morphology and mechanical stability. The only difference between young and 120-days old human RBCs that was reported was a decreased size of old RBCs, produced by a parallel reduction in membrane area and cell volume that guarantees a rather constant surface-to-volume ratio and the maintenance of a biconcave discoid shape (no sphericization). A reduction in deformability was also described, as the result of increased viscosity of the cytoplasm (in turn due to the increase in Hb concentration in a dehydrated cell), not of major alterations in membrane properties. Despite decades of investigation, it is still unclear by which mechanism(s) old RBCs are cleared from the circulation. The most credited hypotheses point to an autoimmune mechanism, and/or the exposure of phosphatidylserine on the outer leaflet of the lipid bilayer as a signal for phagocytosis by macrophages. The issue, however, is still controversial. By a change of perspective, it could be imagined that RBCs are kept in check for their entire life span, by active removal of damaged regions of the membrane, and are sequestered well before they start becoming dysfunctional, perhaps by detection of their reduced size and deformability.

## Posters

**Neuronal corticostriatal VPS13A downregulation induces hyperlocomotion and synaptic plasticity dysfunction in the mouse**

*Esther García-García* ^1,2,3^ *, Sara Conde-Berriozabal* ^1,2,3^ *, Alba Ramón-Lainez* ^1,2,3^ *, Daniel del Toro* ^1,2,3^ *, Albert Giralt* ^1,2,3^ *, Jordi Alberch* ^1,2,3,4^ *, Mercè Masana* ^1,2,3^ *, Manuel J. Rodríguez* ^1,2,3^

^1^ Department of Biomedical Sciences, School of Medicine and Health Sciences, Institute of Neurosciences, Universitat de Barcelona, E-08036 Barcelona, Spain

^2^ August Pi i Sunyer Biomedical Research Institute (IDIBAPS), E-08036 Barcelona, Spain

^3^ Networked Biomedical Research Centre for Neurodegenerative Disorders (CIBERNED), E-08036 Barcelona, Spain

^4^ Production and validation center of advanced therapies (Creatio), Faculty of Medicine and Health Science, University of Barcelona, E-08036 Barcelona, Spain

Chorea-acanthocytosis (ChAc) is a rare autosomal recessive neurodegenerative disorder caused by *VPS13A* gene mutations leading to marked reduction or absence of VPS13A. ChAc patients show adult-onset progressive movement disorders such as chorea, dystonia, parkinsonism, seizures, peripheral neuropathy, and red blood cell acanthocytosis. The main neuropathological feature in ChAc is selective degeneration of the striatum. The study of the VPS13A function in the brain has been poorly addressed due to the lack of appropriate models. Therefore, the objective of this work is [1] to develop a ChAc cell and mouse model using miR30-based shRNA knock-down (KD) system and [2] to evaluate how synaptic plasticity and neuronal function in corticostriatal circuits are affected when VPS13A is down-regulated. We encapsulated two miR30-shRNA against *VPS13A* in an AAV viral vector. To generate a cellular model, we infected primary cortical neurons with both AAV-Ctrl and AAV-KD. AAV-KD primary cortical neurons presented a decrease in the PSD95, p75NTR, and BDNF levels compared with AAV-Ctrl neurons. We then generated an in vivo model by injecting AAV-Ctrl and AAV-KD bilaterally in M2 cortex and in two different regions of the striatum, using stereotaxic surgery in 8-week-old mice. Behavioral tests revealed hyperlocomotion and an increment in exploratory behavior in KD mice compared to Ctrl mice. KD mice presented a decrease in the BDNF levels compared with controls. Electrophysiological recordings showed that KD mice presented impaired striatal LTD after a theta-burst stimulation of cortical afferences, revealing a corticostriatal synaptic plasticity dysfunction in these KD mice. This corticostriatal synaptic dysfunction was partially recovered when we treated the slices with BDNF. These results suggest a role of VPS13A in the control of neuronal plasticity mechanisms. Understanding how the absence of VPS13A modulates synaptic function will contribute to further knowledge of ChAc pathophysiology.

Supported by grant from the Ministerio de Ciencia y Innovación (grant number: PID2020-119386RB-I00) and Fundación ChAc (Spain).

**Suppressor mutations in yeast VPS13 suggest a cooperative mechanism for lipid transport**

*Jae-Sook Park and Aaron Neiman*

Department of Biochemistry and Cell Biology, Stony Brook University, NY, USA

Yeast cells lacking the mitochondrial-ER contact site complex ERMES cannot grow in the absence of glucose due to mitochondrial defects. However, spontaneous suppressors arise that allow growth on non-fermentable carbon sources. These suppressors are caused by point mutations in the yeast *VPS13* gene that, presumably, enhance lipid transfer to the mitochondrion and compensate for the loss of ERMES. Earlier studies suggested that suppression was due to increased localization of the mutant VPS13 protein to mitochondrial membrane contact sites. However, we have found that the same mutation that bypasses the need for ERMES can also partially compensate for defects in VPS13 function at the endosome and at the prospore membrane, which cannot be explained by increased occupancy at mitochondrial contacts. Rather, these results suggest that the mutation makes the mutant VPS13 more efficient at lipid transfer.

More than a dozen different such “suppressor” alleles of *VPS13* have been isolated. These mutations are spread out over a 1500aa amino acid region of the protein comprising most of the RBG repeats that make up the hydrophobic channel. Mapping these mutations onto the Alphafold structure reveals that they can occur in at least seven different RBG repeats (of 12 total) and that they are enriched at positions in the hinge region of the repeat. There is little primary sequence identity between RBG repeats. However, using a structural alignment of different RBG domains allowed us to identify corresponding residues in different repeats. This revealed that several suppressors are located at the same position in different repeats. Moreover, we were able to engineer new suppressor mutations using these alignments. The observation that mutations in a (possibly) flexible hinge region of the RBG repeat gives rise to alleles with enhanced lipid transfer activity suggests that the mechanism of transfer involves a conformational change in the RBG repeat. That single mutations in different RBG repeats produce the same phenotype further suggests that this conformational change involves cooperative movement of the repeats. A model incorporating these observations will be discussed.

**Role of neuronal VPS13A in mitochondria homeostasis and functioning in a model of Chorea-Acanthocytosis**

*A. Ramon-Lainez*^1,2,3*^, *E. García-García*^1,2,3*^, D. del Toro^1,2,3^*, G. Escaramís*^1,4^*, L. López-Molina*^1,2,3^*, L. Valls*^2^*, G. Garrabou*^2^*, E. Martí*^1,4^*, S. Ginés*^1,2,3^*, M. Masana*^1,2,3^*, J. Alberch*^1,2,3^*, MJ. Rodríguez*^1,2,3^

^1^ Department Biomedical Sciences, Institute of Neurosciences, School of Medicine and Health Sciences, University of Barcelona, Spain

^2^ August Pi Sunyer Biomedical Research Institute (IDIBAPS), Barcelona, Spain

^3^ Network Center for Biomedical Research in Neurodegenerative Diseases (CIBERNED), Spain

^4^ Biomedical Research Networking Center for Epidemiology and Public Health (CIBERESP), Spanish Ministry of Science and Innovation, Madrid, Spain

* These authors contributed equally to this work.

Loss-of-function mutations in the human vacuolar protein sorting 13 homolog A (*VPS13A*) gene encoding the VPS13A protein cause Chorea Acanthocytosis (ChAc). ChAc is an ultra-rare disease that causes progressive movement disorders such as chorea and dystonia, but only sympgarciatomatic treatment is currently available. At the cellular level, VPS13A is located in mitochondria-endoplasmic reticulum (ER) contact sites, where it has been proposed to act as a lipid transfer protein. However, the VPS13A role in neuronal functioning remains unknown. To gain insights into its possible functions, in this study, we assessed VPS13A interacting partners in the wild-type mouse brain through a specific protein immunoprecipitation protocol followed by mass spectrometry. We found that VPS13A specifically interacts with different mitochondrial proteins including enzymes of the tricarboxylic acid cycle such as subunits of the mitochondrial isocitrate dehydrogenase complex, and with Mre11a and Rad50. Interestingly, we also found that corticostriatal VPS13A downregulation in vivo reduced the concentration of these proteins. Mre11a and Rad50 together with Nbn as MRN complex acts as a sensor recognizing double-stranded DNA breaks not only in the nucleus but also in mitochondria and cytoplasm. In cortical neuron cultures, we found that VPS13A is located in neuronal MAMs and that its downregulation specifically increases the content of mitochondrial reactive oxygen species, but does not induce neuronal ER-stress. Taken together, these results point to a putative role of VPS13A in the regulation of mitochondrial energy metabolism and consequently, the proper functionality of the neuron. Furthermore, the interaction of VPS13A with proteins of the MRN complex suggests that mitochondrial DNA repair dysfunction may be a possible new pathophysiological mechanism of ChAc that is worth exploring. In conclusion, unveiling the role of VPS13A in the mitochondrial-associated ER membranes and mitochondrial functioning could yield fresh perspectives on the role of VPS13A in neurons and enhance our understanding of the pathophysiology of ChAc.

Supported by the Ministerio de Ciencia e Innovación (grant number: PID2020-119386RB-I00 to JA and MJR, PID2021-123732OB-I00 to SG, PID2021-124896OA-I00 to MM) and Fundación ChAc (Spain).

**Epilepsy in VPS13A disease: a retrospective evaluation of EEG and clinical data in a cohort of ten patients**

*Paul Šari*ć^1^, *Kevin Peikert*^2,3,4^*, Valentin Neubert*^1^*, Andreas Hermann*^2,3,5^*, Timo Kirschstein*^1,3,6^

^1^ Oscar Langendorff Institute for Physiology, University Medical Centre Rostock, University of Rostock, Germany

^2^ Translational Neurodegeneration Section “Albrecht-Kossel”, Department of Neurology, University Medical Centre Rostock, University of Rostock, Germany

^3^ Centre for Transdisciplinary Neurosciences Rostock (CTNR), University Medical Centre Rostock, Germany

^4^ United Neuroscience Campus Lund-Rostock (UNC), Rostock site

^5^ Deutsches Zentrum für Neurodegenerative Erkrankungen (DZNE) Rostock/Greifswald, Rostock, Germany

^6^ Department of Neurology, University Medical Centre Rostock, University of Rostock, Germany

**Background:** VPS13A disease (formerly known as chorea-acanthocytosis) is a rare autosomal-recessive neurodegenerative disorder caused by mutations in the *VPS13A* gene, which presents with a Huntington’s disease-like phenotype. Typical manifestations consist of different movement disorders (like chorea, dystonia, tics and parkinsonism), dysarthria and dysphagia, behavioural changes as well as neuromuscular involvement. Furthermore, epilepsy is observed in almost half of affected individuals: Seizures may occur during the course of the disease but can also be the first manifestation.

**Aims:** To investigate prevalence and characteristics of epilepsy in VPS13A disease patients.

**Methods:** We retrospectively studied electroencephalography (EEG) and clinical data in a cohort of ten VPS13A disease patients from two centres in Germany (mean age 43.1 years, two females and eight males). EEGs were evaluated qualitatively and annotated for perspective quantitative analysis using the EDF browser. Patient-files were reviewed to correlate the patients’ history with the collected EEG-data.

**Results:** In total, 64 EEGs of seven patients were analysed, of which 62 were found to have at least one pathological aspect. The remainder without pathological findings were derived from a single patient without epilepsy. Pathological changes mostly consisted of regional theta and delta slowing mainly recorded from temporal and occipital leads. They were longitudinally stable. Six patients showed generalised slowing with three cases harbouring theta rhythms as the dominating frequency. One of those having a normal Berger-effect, even showed a theta background rhythm. Epileptiform discharges (EDs) were present in one patient, who also had frontal intermittent rhythmic delta activity (FIRDA) in several recordings. FIRDA was also present in a further patient, who additionally showed frontal intermittent rhythmic theta activity (FIRTA).

Clinical data from nine patients were reviewed. All but one (89%) suffered from epilepsy, of whom 25% had a seizure as first manifestation of the disease. Mean age at onset of VPS13A disease was 22.2 years. Mean age at onset of seizures was 25.4 years. The seizures presented mostly as (secondarily) generalised tonic-clonic seizures and focal seizures with unconsciousness. Seizure frequency ranged from once a year and less up to two times a week. The number of anticonvulsants administered ranged from two to four. Hence, drug resistant epilepsy was detected in 75% of the patients. In two of our cases, temporal lobe epilepsy was previously reported (Scheid *et al*., 2009, Neurology). One of these patients was subsequently prescribed eight anticonvulsants, either in mono or combination therapy. A partial temporal lobe resection in this patient failed to have any long-term effect. Two patients died suddenly unexplained.

**Conclusion:** Epilepsy is very common and manifests early in VPS13A disease. It may occur even more frequently as previously reported. Of note, we found a high proportion of drug-resistance in our cohort implicating that epilepsy significantly contributes to disease severity and burden. EEG-data show a broad variety of pathological changes (e.g., regional and generalised slowing FIRDA, FIRTA, EDs). This study highlights, that *VPS13A* should be considered a genetic cause of epilepsy, particularly, but not necessarily, if additional symptoms such as movement disorders, neuromuscular involvement or cognitive-psychiatric disorders are present.

**An autopsy series of seven cases of VPS13A disease**

*Ricky M. Ditzel Jr., BSHS* ^1,2,3,4,5^ *, Ruth H. Walker, MB, ChB, PhD* ^6,7^ *, Melissa J. Nirenberg, MD, PhD* ^6,7^ *, Amber M. Tetlow, PhD* ^1,2,3,4,5^ *, Kurt Farrell, PhD* ^1,2,3,4,5^ *, Emma L. Thorn MA* ^1,2,3,4,5^ *, Diana K. Dangoor, BA* ^1,2,3,4,5^ *, Ronald Gordon, PhD* ^1^ *, Claudia De Sanctis, PhD* ^1,2,3,4,5^ *, Antonio Velayos-Baeza, PhD* ^8,9^ *, Gabriel Miltenberger-Miltenyi, MD, PhD* ^10,11,12,13^ *, Jack Humphrey, PhD* ^3,4,6,14^ *, John F. Crary, MD PhD* ^1,2,3,4,5^

^1^ Department of Pathology, Molecular, and Cell Based Medicine, Icahn School of Medicine at Mount Sinai, New York, New York, USA

^2^ Department of Artificial Intelligence & Human Health, Icahn School of Medicine at Mount Sinai, New York, New York, USA

^3^ Nash Family Department of Neuroscience, Icahn School of Medicine at Mount Sinai, New York, NY, USA

^4^ Friedman Brain Institute, Ronald M. Loeb Center for Alzheimer’s Disease, Icahn School of Medicine at Mount Sinai, New York, New York, USA

^5^ Neuropathology Brain Bank & Research CoRE, Icahn School of Medicine at Mount Sinai, New York, New York, USA

^6^ Department of Neurology, Icahn School of Medicine at Mount Sinai, New York, New York, USA

^7^ James J. Peters Veterans Affairs Medical Center, Bronx, NY, USA

^8^ Department of Physiology, Anatomy, and Genetics, University of Oxford, UK

^9^ Welcome Centre for Human Genetics, University of Oxford, Oxford, UK

^10^ Laboratório de Genética, Faculdade de Medicina, Universidade de Lisboa, Lisbon, Portugal

^11^ Department of Neurology, Ludwig-Maximilians-Universität München, Munich, Germany

^12^ Reference Center on Lysosomal Storage Diseases, Hospital Senhora da Oliveira, Guimarães, Portugal

^13^ Instituto de Medicina Molecular João Lobo Antunes, Faculdade de Medicina da Universidade de Lisboa, Lisbon, Portugal

^14^ Department of Genetics and Genomic Sciences & Icahn Institute for Data Science and Genomic Technology, Icahn School of Medicine at Mount Sinai, New York, NY, USA

Vacuolar protein sorting 13 homolog A (VPS13A) disease, historically known as chorea-acanthocytosis, is a rare neurodegenerative disorder caused by biallelic mutations in *VPS13A*, resulting in reduced or absent VPS13A protein. Neuropathological features include neuronal loss, most prominently in the caudate nucleus, with associated marked astrogliosis. There are no known disease-specific cellular changes (e.g., protein aggregation). VPS13A localizes to contact sites between subcellular organelles, consistent with its recently-identified role in lipid transfer between membranes. To date, autopsy reports have been limited, often lacking genetic or biochemical diagnostic confirmation. In this study brain tissues, clinical data, and other diagnostic data from 7 clinically typical cases of VPS13A disease were collected from contributing centers. Tissues underwent routine, special, and immunohistochemical staining focused on neurodegeneration. Electron microscopy was performed in one case. Immunoblots confirmed the loss of VPS13A protein in some cases, and sequencing identified VPS13A mutations in all cases, including novel variants. Gross examination showed severe striatal atrophy. Microscopically, there was neuronal loss and astrogliosis in affected regions. Luxol Fast Blue staining showed variable lipid accumulations with diverse morphologies, a novel finding that was confirmed by electron microscopy. Two cases had comorbid Alzheimer’s neuropathologic changes; one had brainstem-predominant Lewy body pathology in the absence of clinical Parkinsonism. We also noted the variable presence of rare degenerating p62- and ubiquitin-positive cells in affected regions. Calcifications were present in three cases, being extensive in one. We present the largest autopsy series of biochemically- and genetically-confirmed VPS13A disease and report a detailed survey of histopathological findings.

**Red blood cell shape during flow as a biomarker for Neuroacanthocytosis syndromes in comparison to Huntington’s disease**

*Marcelle G. M. Lopes* ^1,2^ *, Steffen M. Recktenwald* ^1^ *, Alzbeta Mühlbäck* ^3,4,5^ *, Kevin Peikert* ^6,7,8^ *, Andreas Hermann* ^6,7,8^ *, Stephan Quint* ^2^ *, Christian Wagner* ^1,9^ *, Lars Kaestner* ^1,10^

^1^ Experimental Physics, Saarland University, Saarbruecken, Germany

^2^ Cysmic GmbH, Saarbruecken, Germany

^3^ Department of Neurology, Ulm University, Ulm, Germany

^4^ Huntington Center South, Taufkirchen, Germany

^5^ Department of Neurology and Center of Clinical Neuroscience, Charles University and General University Hospital in Prague, Prague, Czechia

^6^ Translational Neurodegeneration Section “Albrecht Kossel”, University of Rostock, Rostock, Germany

^7^ Department of Neurology, University of Rostock, Rostock, Germany

^8^ Center for Transdisciplinary Neurosciences Rostock (CTNR), University of Rostock, Rostock, Germany

^9^ Physics and Materials Science Research Unit, University of Luxembourg, Luxembourg, Luxembourg

^10^ Theoretical Medicine and Biosciences, Saarland University, Homburg/Saar, Germany

The deformability of red blood cells (RBC), allowing them to adjust their shape according to the vessel’s flow conditions, is crucial for microvascular perfusion and gas exchange. However, this adaptability is compromised in specific diseases, such as Neuroacanthocytosis syndromes (NAS). The NAS spectrum includes the neurodegenerative disorders chorea-acanthocytosis (VPS13A disease) and McLeod syndrome (XK disease) caused by gene mutations in *VPS13A* and *XK*, respectively. They are phenotypically very similar to HD, where CAG repeat expansions in the *HTT* gene lead to neurodegeneration. While some RBC abnormalities have been equally described in HD, acanthocytes typically do not occur.

Diagnosing NAS is complicated, frequently delayed, and primarily relies on identifying the gene mutations through genetic testing. To address this challenge, we propose utilizing the mechanical properties of RBC during flow as a biomarker for monitoring drug response in clinical studies of NAS and HD. Beyond improving diagnostic accessibility, comprehensive disease monitoring offers a pathway to gaining a deeper understanding of disease progression and potential therapeutic avenues.

In this context, we employed the “Erysense” technology, to assess the morphology of the RBCs during microcapillary flow in patients with NAS and HD. The “Erysense” platform replicates microvascular blood flow within microchannels of a size similar to that of RBCs. An algorithm based on artificial neural networks is utilized to characterize RBC shapes, distinguishing between healthy and pathological single-cell shapes. Subsequently, a morphological report is generated for the samples, providing the potential for diverse diagnostic evaluations. The investigation encompassed 9 NAS patients (4 VPS13A; 5 XK), 7 HD patients and 6 control samples.

The approach using “Erysense” offered a clear distinction between healthy controls and patients with both diseases. This distinction becomes evident through shape variations, as we identified acanthocytes in NAS patients (including both XK disease and VPS13A disease) and recognized RBCs with compromised membranes in HD samples. Additionally, it extended to equilibrium flow positions under varying flow conditions.

Given that these diseases result in substantial yet systematic and reproducible alterations in RBC morphology during capillary flow, we have demonstrated that the observed morphological characteristics in the capillary flow of RBCs can be a functional biomarker for monitoring both NAS and HD, as well as monitoring drug response in clinical studies.

**Stimulated emission depletion (STED) microscopy detects actin and spectrin distribution in acanthocytes compared to healthy red blood cells**

*Min Qiao* ^1,2^ *, Ahmad Lotfinia* ^3^ *, Kevin Peikert* ^4,5,6^ *, Andreas Hermann* ^4,5,6^ *, Greta Simionato* ^1,7^ *, Thomas John* ^1^ *, Marcel Lauterbach* ^3^ *, Lars Kaestner* ^1,2^

^1^ Experimental Physics, Saarland University, Saarbrücken, Germany

^2^ Theoretical Medicine and Biosciences, Saarland University, Homburg, Germany

^3^ Center for Integrative Physiology and Molecular Medicine, Saarland University, Homburg, Germany

^4^ Translational Neurodegeneration Section “Albrecht Kossel”, Department of Neurology, University Medical Center Rostock, University of Rostock, Rostock, Germany

^5^ Department of Neurology, University Medical Center Rostock, University of Rostock, Rostock, Germany

^6^ Center for Transdisciplinary Neurosciences Rostock (CTNR), University Medical Center Rostock, University of Rostock, Rostock, Germany

^7^ Institute for Clinical and Experimental Surgery, Saarland University, Campus University Hospital, Homburg, Germany

Neuroacanthocytosis syndromes (NAS) refer to a group of inherited neurodegenerative disorders, including VPS13A disease (chorea-acanthocytosis) and XK disease (McLeod syndrome). Acanthocytes are characterized by a few irregular spicules projected from the surface of the membrane. Föller *et al*. (FASEB J. 2012) described depolymerized actin in acanthocytes. Whether there are differences of cytoskeleton properties or distribution between healthy RBCs and acanthocytes is still elusive.

Stimulated emission depletion (STED) microscopy belongs to the super-resolution microscopy. It uses a pair of lasers to control the excitation state of fluorescent molecules in a targeted manner to resolve details, which are separated far less than the wavelength of the observation light. This technique can provide more details of the cell ultrastructure, including the cytoskeleton. We used this technique to detect and quantify the actin and spectrin distribution of acanthocytes compared to healthy control RBCs.

RBCs from 2 healthy donors and NAS patients were collected one day before experiments (3 VPS13A, 1 XK). Microscopy slides were precoated with cell-tak. Then washed RBCs suspensions were placed on the coated slides for sedimentation. For actin staining, RBCs were fixed with 0.1% glutaraldehyde (GA) for 10 minutes. Then cells were permeabilized with 0.5% triton X-100 for 10 minutes followed by staining with 3% phalloidin 647N for 30 minutes. For spectrin staining, RBCs were first fixed with 0.1% GA and 3% paraformaldehyde for 10 minutes. Permeabilization of the RBCs and blocking of unspecific binding sites was done simultaneously by adding 3% bovine serum albumin and 0.05% trition X-100 for 1 hour. Then cells were stained with the primary spectrin antibody for 2 hours at room temperature. After that, cells were loaded with the secondary (fluorescent) antibody for 1 hour in the dark at room temperature. After staining, microscopy slides with RBCs were sealed with mowiol as a mounting solution. The prepared slides were imaged by confocal and STED microscopy within 1 week. RBCs from healthy controls as well as acanthocytes and discocytes from patients were recorded for detecting actin and spectrin distribution separately. We could not find obvious structural differences as Föller *et al*. described in the actin and spectrin distributions of acanthocytes compared to healthy RBCs.
